# Supplementary material for: Penta- and hexa-coordinated beryllium and phosphorus in high-pressure modifications of CaBe2P2O8
Source: Nat Commun. 2019 Jun 26;10:2800. doi: 10.1038/s41467-019-10589-z (PMC6594954; doi:10.1038/s41467-019-10589-z)
Supplement: Supplementary file 1 — Supplementary information [file 41467_2019_10589_MOESM1_ESM.pdf]

Supplementary information

for the manuscript “Penta- and hexa-coordinated beryllium and phosphorus in high-pressure modifications of $CaBe_2P_2O_8$”

by Anna Pakhomova et al.

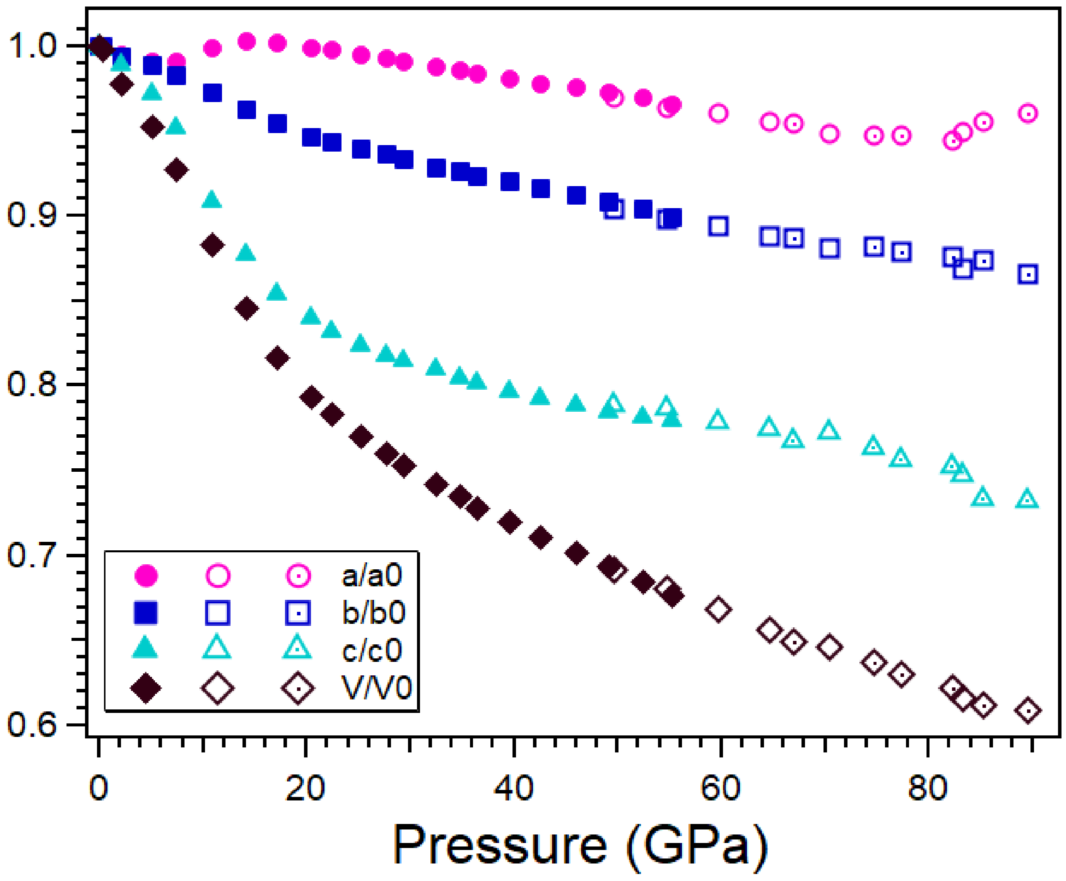

Supplementary Figure 1. High-pressure evolution of normalized unit-cell parameters of hurlbutite, $CaBe_2P_2O_8$. Filled, open and open with dots symbols correspond to experiments **1**, **2** and **3**, respectively. The errors are smaller than the size of the symbols.

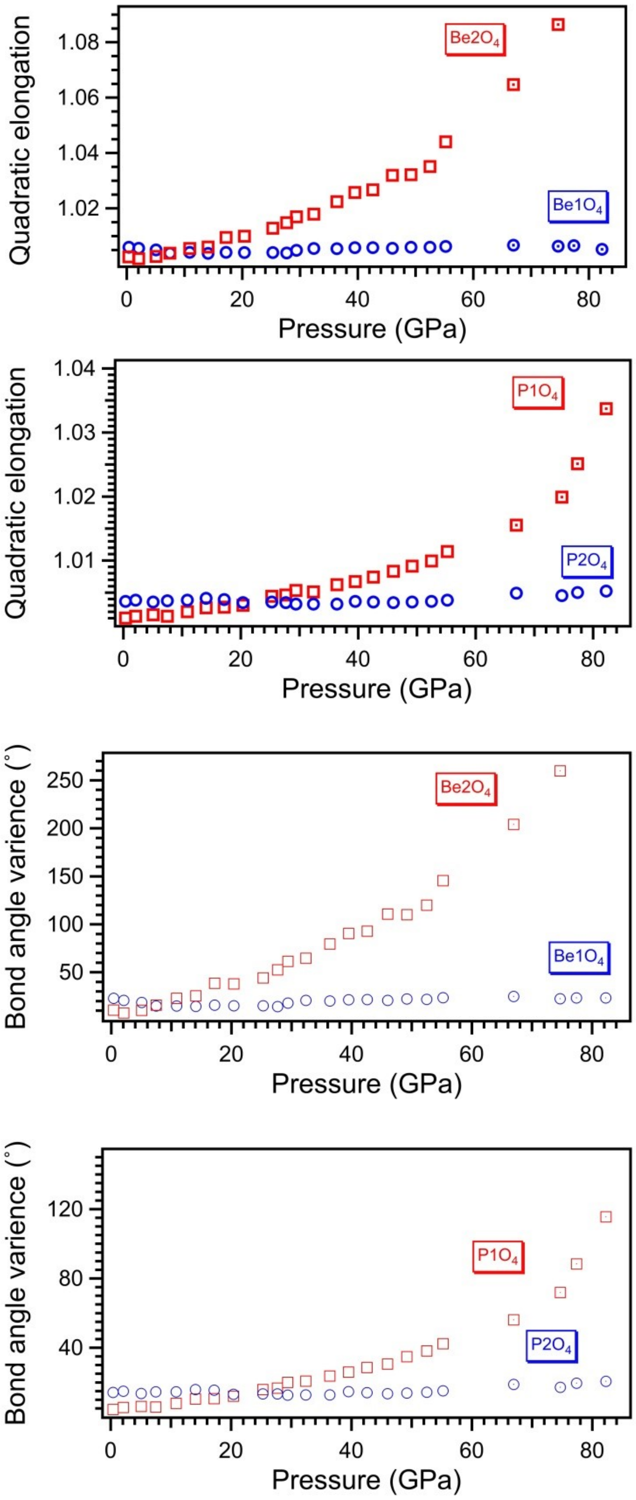

Supplementary Figure 2. The evolution of quadratic elongation (QE) and bond angle variance (BAV,°) of PO_4 and BeO_4 tetrahedra along the compression of hurlbutite, $CaBe_2P_2O_8$. The parameters QE and BAV that show deviation of TO_4 polyhedra from geometry of ideal tetrahedron are defined as:

QE $= \frac{1}{4}\sum_{i=1}^{4}\left(\frac{l_i}{l_0}\right)^2$ and BAV $= \sqrt{\frac{1}{5}\sum_{i=1}^{6}(\theta_i - 109.47)^2}$ where l_0 is a center-to-vertex distance for ideal tetrahedron whose volume is equal to that of the distorted tetrahedron with bond lengths l_i and bond angles θ_i.

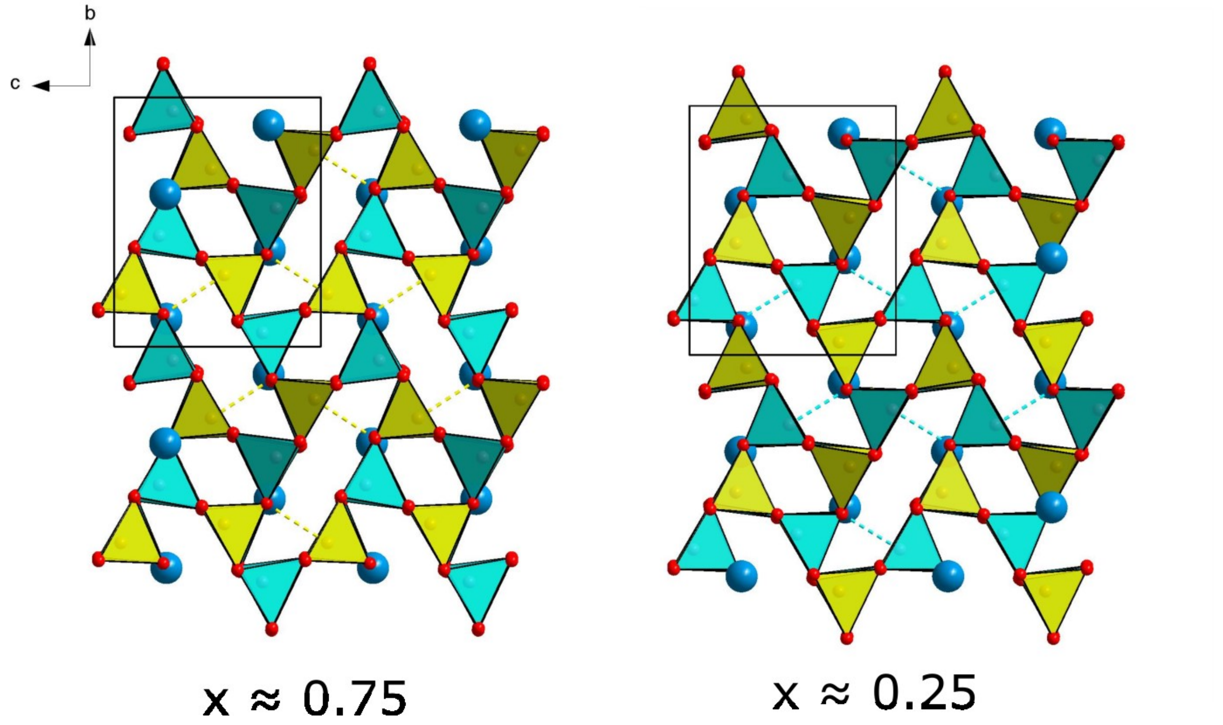

Supplementary Figure 3. The crystal structure of hurlbutite, $CaBe_2P_2O_8$, at 70.3(1) GPa. The dashed yellow and blue lines respectively represent the P1-O8* and Be2-O2* contact that becomes shortened upon pressure increase and accompanying closure of 8-member rings. The current values of P1-O8* and Be2-O2* contacts are 2.26(2) and 2.11(4) Å, respectively. Black solid line outlines a unit cell.

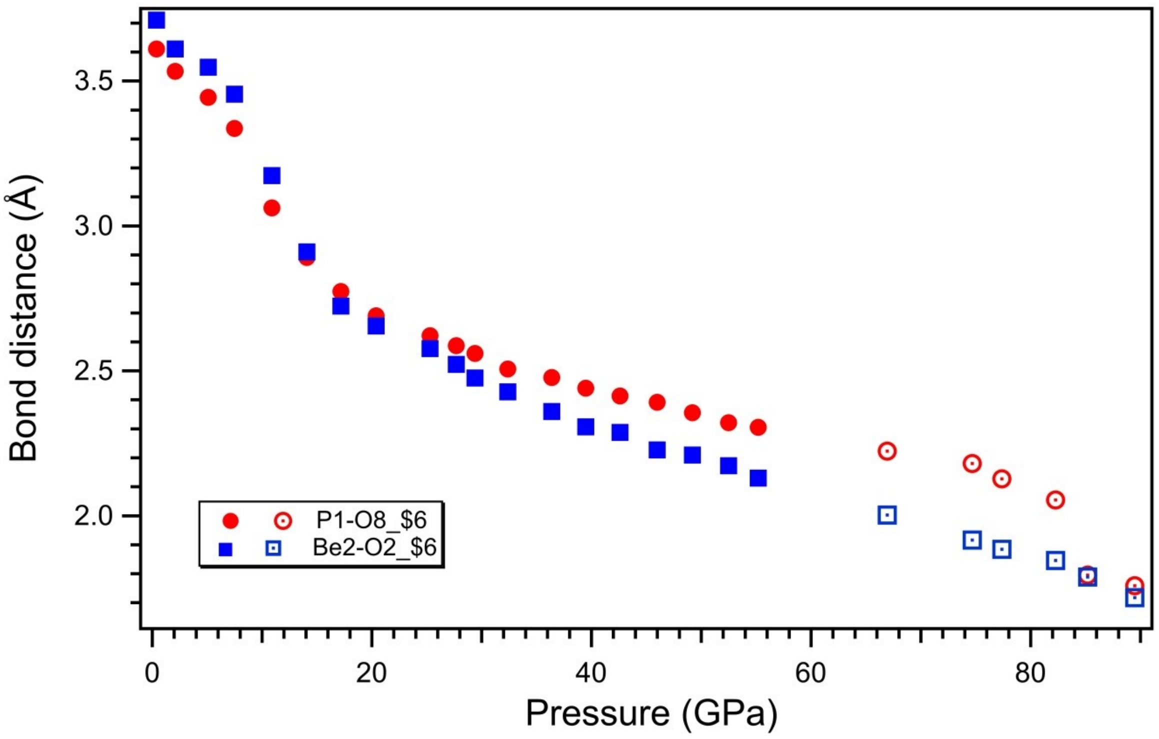

Supplementary Figure 4. Evolution of P1-O8* and Be2-O2* bonds showing the pressure-induced approach of O8* and O2* oxygen atoms to the P1 and Be2 atoms, respectively.

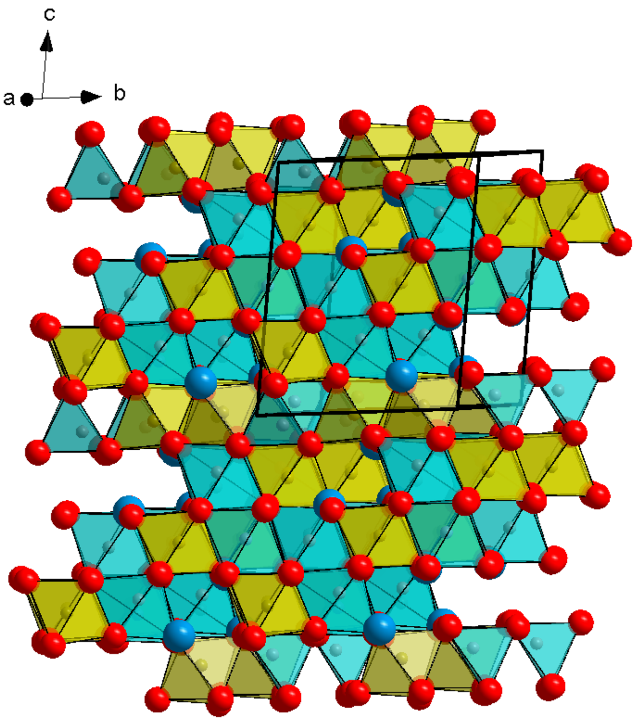

Supplementary Figure 5. Close packing of Ca (blue spheres) and O (red spheres) in the crystal structure of hurlbutite-IV, $CaBe_2P_2O_8$. BeO_6 and PO_6 octahedra are given in blue and yellow, respectively. Black solid line outlines a unit cell.

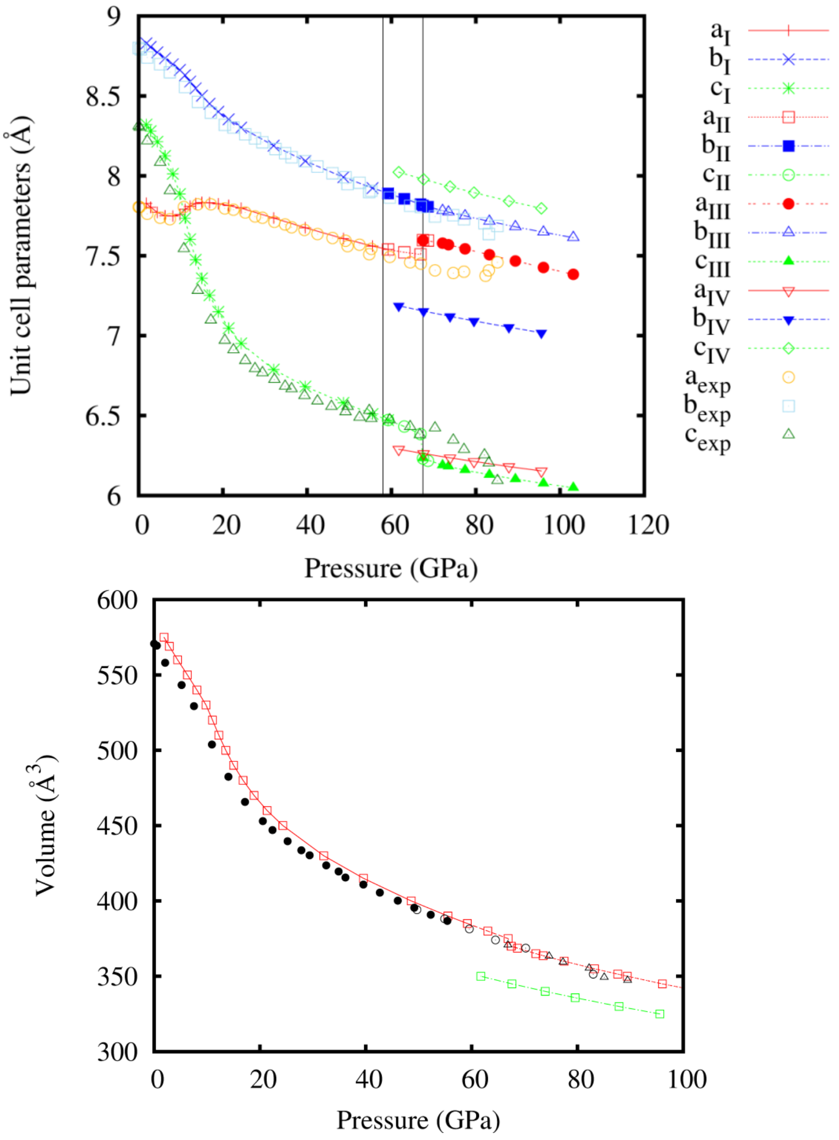

Supplementary Figure 6. Comparison of theoretically calculated and experimentally measured evolution of the unit-cell parameters (a) and volume (b) of hurlbutite, $CaBe_2P_2O_8$, along the compression. In (a) The parameters a_{I-IV}, b_{I-IV}, c_{I-IV} corresponds to theoretically calculated unit-cell parameters of phases I, II, III and IV; the subscript exp denotes the experimental data. Vertical lines correspond to theoretically estimated transition pressures. In (b) the experimental equation of state is shown with black symbols. Filled circles, open circles and open triangles correspond to experiments **1**, **2** and **3**, respectively. Open squares denote *ab initio* data: red symbols correspond to phases I-III and green symbols corresponds to phase IV. Note that the theoretical data for phases I, II and III follow each other smoothly, indicating the continuous transition between phases I → II → III.

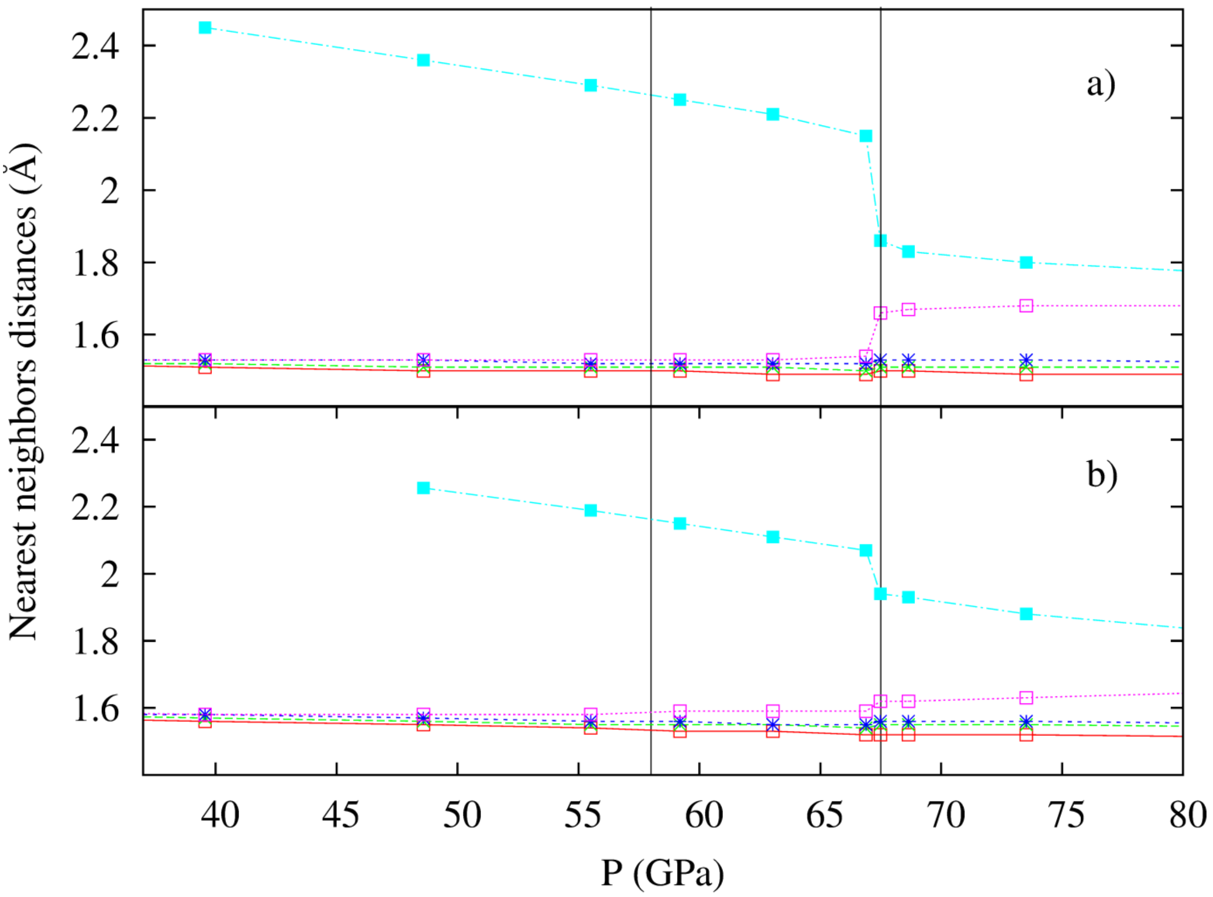

Supplementary Figure 7. Calculated evolution of P-O (a) and Be-O (b) for five nearest neighbor distances in hurlbutite, $CaBe_2P_2O_8$, along the compression. Cyan lines with filled squares represent evolution of P1-O8* (a) and Be2-O2* (b) bond lengths (same as experimental data in Supplementary Figure 4). In calculations we also observed a smooth decreasing of P1-O8* and Be2-O2* bonds up to 67.5 GPa. At this pressure there is a slight drop of Be2-O2* bond length and more pronounced one for P1-O8* indicating the hurlbutite-II to hurlbutite-III transition. Red, blue, green and magenta lines represent the evolution of P-O (a) and Be-O (b) bonds-lengths of initial oxygen tetrahedron. The points almost coincide up to 58 GPa (b) and then start to deviate from each other (in agreement with experimental data in Supplementary Tables 1-2) indicating the transition to hurlbutite-II.

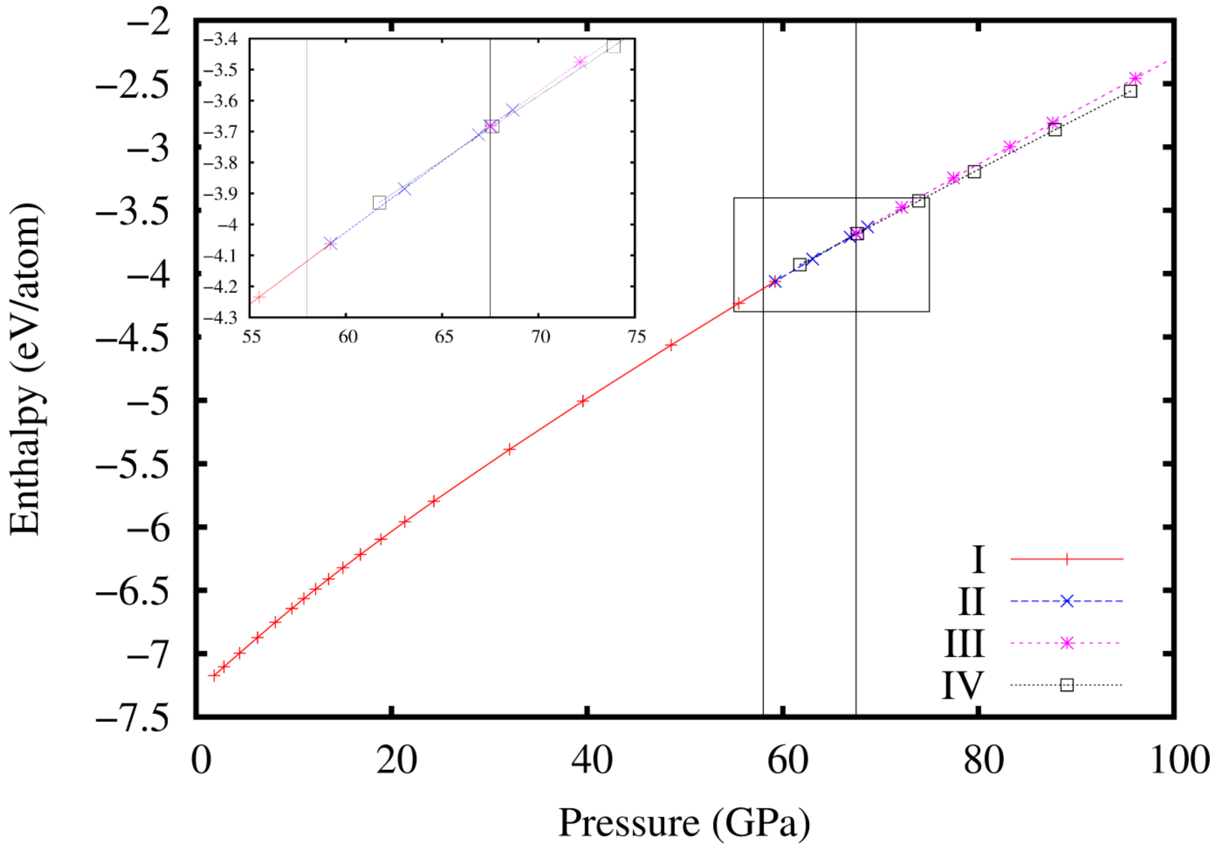

Supplementary Figure 8. Calculated pressure dependence of enthalpy of phases I-IV of hurlbutite, $CaBe_2P_2O_8$. Inset magnifies the image near the phase transitions. One sees that the enthalpies of phases I, II and III follow each other continuously because of the continuous transition between phases I → II → III. Phase IV correspond to a different energy minimum, and its enthalpy is shown with a separate curve, which cross the enthalpy curve of phase II at approximately 65 GPa, which almost coincides with the pressure for hurlbutite-II to hurlbutite-III transition. At higher pressures phase IV has a lower enthalpy, and therefore it is predicted to be the ground state. Phase III is predicted to be metastable at all pressures of its existence.

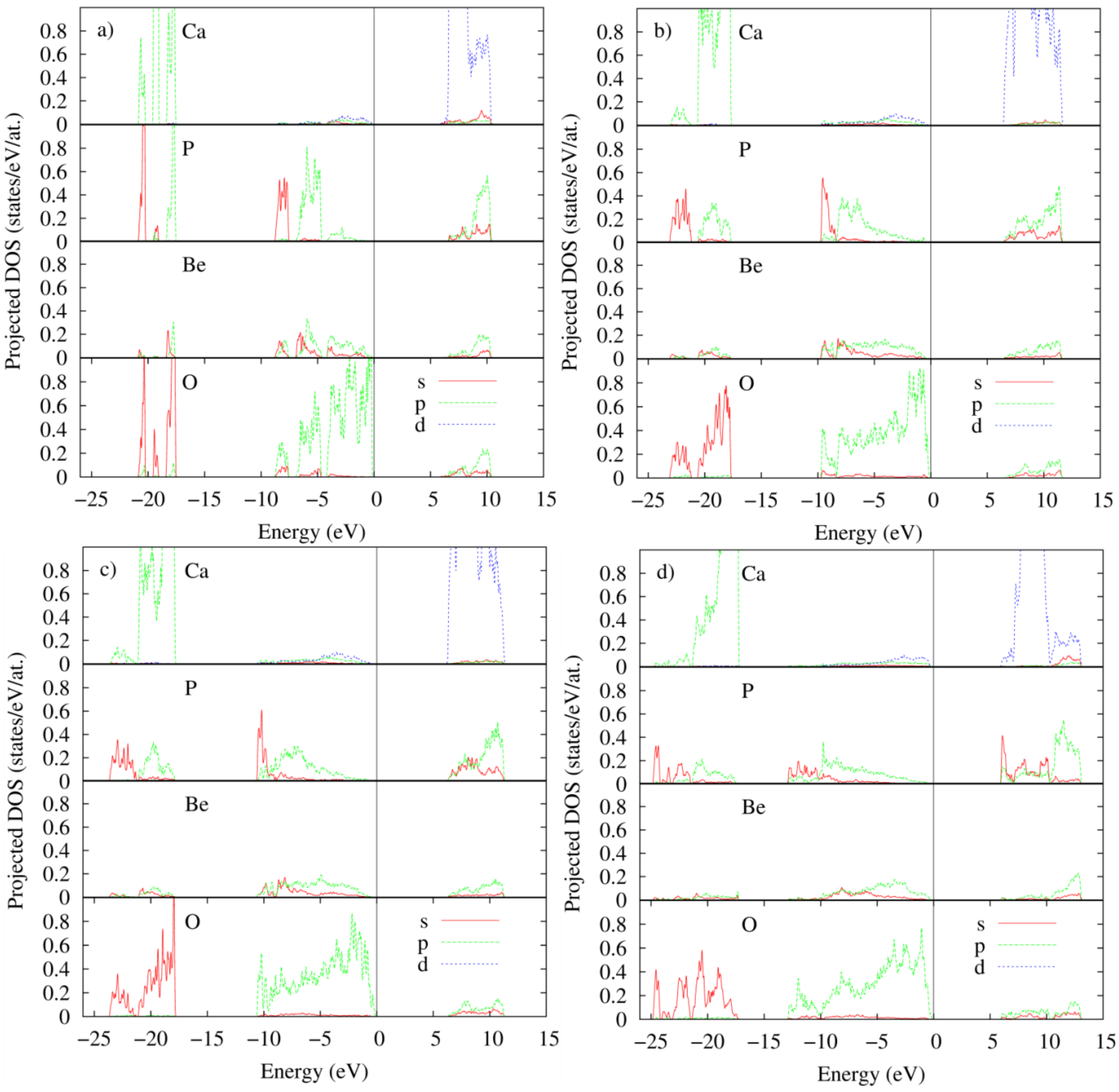

Supplementary Figure 9. Calculated partial local density of electronic states of different phases of hurlbutite, $CaBe_2P_2O_8$, along the compression: (a) hurlbutite at 2.9 GPa; (b) hurlbutite-II at 66.9 GPa; (c) hurlbutite-III at 68.7 GPa; (d) hurlbutite-IV at 79.6 GPa. The pressures correspond to those in Figure 5 of the main text. Energy is given relative to the energy of the highest occupied state. Very small traces of DOS at Ca atoms in the energy interval between -10 eV and 0 are the typical projection artefacts, and physically correspond to O *p*-orbitals leaked into Ca atomic sphere.

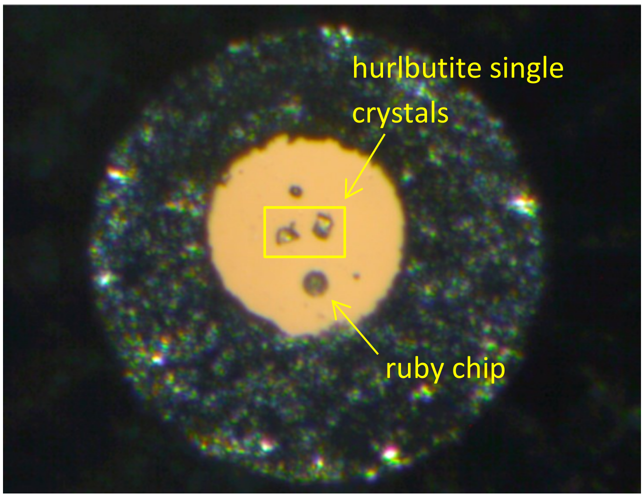

Supplementary Figure 10. Sample chamber loaded with Ne gas in the experiment #**1.**

Supplementary Table 1. Crystallographic data and refinement parameters for hurlbutite polymorphs, $CaBe_2P_2O_8$.

Crystal data	0.4(1) GPa	70.3(1) GPa	74.5(1) GPa	83.2(1) GPa	89.5(1) GPa
	Hurlbutite		Hurlbutite-II	Hurlbutite-III	Hurlbutite-IV
Space group	$P2_1/c$	$P2_1/c$	$P2_1/c$	$P2_1/c$	P-1
a, Å	7.80(2)	7.408(4)	7.3958(15)	7.410(3)	6.203(3)
b, Å	8.7878(4)	7.7456(8)	7.7530(6)	7.6388(8)	7.163(7)
c, Å	8.301(8)	6.425(16)	6.3431(6)	6.209(14)	7.865(3)
α, °	90	90	90	90	80.06(5)
β, °	90.49(18)	89.07(13)	89.136(16)	89.27(12)	85.24(4)
γ, °	90	90	90	90	77.57(6)
Volume, $Å^3$	569.2(18)	368.6(9)	363.67(9)	351.4(8)	335.8(4)
Z	4	4	4	4	4
Data collection					
Wavelength, Å	0.2904	0.2905	0.2907	0.2906	0.2907
Max. θ°	18.487	13.945	15.030	15.311	13.123
Index ranges	$-11\le h\le 12$	$-10\le h\le 11$	$-9\le h\le 9$	$-11\le h\le 13$	$-8\le h\le 8$
	$-16\le k\le 14$	$-12\le k\le 12$	$-11\le k\le 12$	$-13\le k\le 13$	$-6\le k\le 5$
	$-11\le l\le 12$	$-4\le l\le 12$	$-10\le l\le 10$	$-4\le l\le 6$	$-11\le l\le 10$
No.meas.refl.	2304	1150	1175	1142	734
No.uniq.refl.	1203	490	684	558	487
No. obs.refl ($I > 2\sigma(I)$)	1073	416	630	468	396
	Refinement of the structure				
No.of variables	68	68	68	68	115
R_{int}	0.0274	0.0307	0.0265	0.0410	0.0299
R_σ	0.0328	0.0385	0.0227	0.0526	0.0508
R_1, $I > 2\sigma(I)$	0.0370	0.0834	0.0741	0.0681	0.0937
R_1, all data	0.0411	0.0978	0.0711	0.0769	0.1088
wR_2, $I > 2\sigma(I)$	0.0924	0.2321	0.2115	0.1927	0.2464
wR_2, all data	0.0970	0.2718	0.2005	0.2057	0.2802
GooF	1.067	1.178	1.111	1.068	1.201

Supplementary Table 2. Bond distances and polyhedral parameters in hurlbutite polymorphs, $CaBe_2P_2O_8$.

**Pressure**	**0.4(1) GPa**	**70.3(1) GPa**	**74.7(1) GPa**	**83.2(1) GPa**
**Phase**	**Hurlbutite**		**Hurlbutite-II**	**Hurlbutite -III**
	$P1O_4$			***$P1O_5$***
**P1-O6**	1.509(6)	1.518(19)	1.479(4)	1.475(9)
**P1-O3**	1.536(4)	1.500(7)	1.483(6)	1.498(4)
**P1-O8**	1.541(4)	1.47(2)	1.513(4)	1.674(15)
**P1-O1**	1.548(2)	1.489(8)	1.487(3)	1.504(7)
**P1-O8***	3.610(4)	2.25(57)	2.180(4)	1.801(14)
**< P1-O>**	1.533	1.493	1.491	1.590
**Volume**	1.8473	1.6685	1.651	3.3208
	$P2O_4$			
**P2-O2**	1.524(2)	1.496(8)	1.477(3)	1.470(4)
**P2-O4**	1.531(3)	1.459(18)	1.506(4)	1.525(14)
**P2-O7**	1.541(6)	1.532(18)	1.505(3)	1.475(11)
**P2-O5**	1.542(3)	1.484(7)	1.472(6)	1.475(4)
< P2-O>	1.534	1.493	1.490	1.4855
Volume	1.8439	1.6893	1.686	1.6684
	$Be1O_4$			
**Be1-O8**	1.608(3)	1.469(16)	1.467(6)	1.481(7)
**Be1-O3**	1.613(6)	1.53(4)	1.559(8)	1.52(2)
**Be1-O1**	1.627(9)	1.55(4)	1.515(7)	1.53(2)
**Be1-O7**	1.635(6)	1.525(14)	1.559(12)	1.533(8)
<Be1-O>	1.620	1.518	1.525	1.516
Volume	2.165	1.781	1.804	1.762
	$Be2O_4$		***$Be2O_5$***	
**Be2-O6**	1.592(6)	1.504(13)	1.496(13)	1.487(7)
**Be2-O4**	1.623(8)	1.58(4)	1.533(9)	1.504(16)
**Be2-O5**	1.641(3)	1.52(2)	1.524(7)	1.548(14)
**Be2-O2**	1.641(4)	1.55(4)	1.704(10)	1.72(2)
**Be2-O2***	3.711(7)	2.11(4)	1.916(9)	1.79(2)
<Be2-O>	1.624	1.540	1.635	1.610
Volume	2.190	1.754	3.583	3.456
	CaO_7	***CaO_{11}***	***CaO_{11}***	***CaO_{11}***
**Ca-O3**	2.414(3)	2.426(8)	2.374(3)	2.287(17)
**Ca-O5**	2.444(3)	2.258(15)	2.241(3)	2.212(9)
**Ca-O1**	2.446(3)	2.250(7)	2.248(5)	2.170(5)
**Ca-O4**	2.458(4)	2.416(14)	2.327(6)	2.251(11
**Ca-O8**	2.474(7)	2.359(13)	2.332(5)	2.360(7)
**Ca-O7**	2.474(5)	2.24(2)	2.247(4)	2.217(6)
**Ca-O2**	2.581(6)	2.410(11)	2.445(5)	2.468(7)
**Ca-O1***	*3.092(7)*	-	-	-
**Ca-O5***	3.206(8)	-	-	-
**Ca-O6**	-	2.262(12)	2.268(3)	2.248(8)
**Ca-O7**	-	2.304(9)	2.287(3)	2.255(14)
**Ca-O3**	-	2.45(2)	2.437(5)	2.459(6)
**Ca-O4**	-	2.520(13)	2.571(3)	2.538(10)
<Ca-O>^{7}	2.470	2.354	2.343	2.315
Volume	19.978	28.886	28.564	27.621

Supplementary Table 3. Bond distances and polyhedral parameters in hurlbutite-IV, $CaBe_2P_2O_8$, at 89.5(1) GPa.

$P1O_6$		$P2O_6$		$P3O_6$		$P4O_6$	
**P1-O6**	1.57(1)	**P2-O2**	1.50(1)	**P3-O10**	1.55(1)	**P4-O12**	1.48(3)
**P1-O3**	1.59(2)	**P2-O14**	1.50(3)	**P3-O8**	1.63(2)	**P4-O4**	1.61(1)
**P1-O4**	1.60(2)	**P2-O11**	1.68(3)	**P3-O1**	1.64(2)	**P4-O5**	1.61(2)
**P1-O13**	1.69(3)	**P2-O8**	1.64(2)	**P3-O1**	1.66(1)	**P4-O16**	1.63(2)
**P1-O15**	1.69(3)	**P2-O13**	1.68(1)	**P3-O9**	1.63(3)	**P4-O16**	1.71(2)
**P1-O11**	1.77(1)	**P2-O7**	1.72(2)	**P3-O15**	1.75(3)	**P4-O7**	1.76(2)
<P1-O>	1.65	***<P2-O>***	1.62	***<P3-O>***	1.64	***<P3-O>***	1.63
Volume	5.93	***Volume***	5.60	***Volume***	5.88	***Volume***	5.69
BAV	35.81	***BAV***	24.16	***BAV***	7.84	***BAV***	46.35
QE	1.0125	***QE***	1.0105	***QE***	1.0040	***QE***	1.0161
$Be1O_6$		$Be2O_6$		$Be3O_6$		$Be4O_6$	
**Be1-O2**	1.58(2)	**Be2-O10**	1.51(3)	**Be3-O6**	1.43(7)	**Be4-O6**	1.52(4)
**Be1-O15**	1.62(3)	**Be2-O11**	1.63(4)	**Be3-O12**	1.68(4)	**Be4-O5**	1.61(6)
**Be1-O8**	1.65(6)	**Be2-O9**	1.66(8)	**Be3-O15**	1.68(2)	**Be4-O14**	1.61(3)
**Be1-O11**	1.66(3)	**Be2-O16**	1.72(3)	**Be3-O7**	1.71(3)	**Be4-O14**	1.75(4)
**Be1-O3**	1.74(6)	**Be2-O4**	1.74(3)	**Be3-O10**	1.91(6)	**Be4-O13**	1.75(3)
**Be1-O3**	1.81(2)	**Be2-O7**	1.79(7)	**Be3-O2**	1.81(3)	**Be4-O7***	2.09(4)
<Be1-O>	1.68	***<Be2-O>***	1.67	***<Be3-O>***	1.70	***<Be4-O>***	1.65/1.72*
Volume	6.21	***Volume***	6.05	***Volume***	6.46	***Volume***	3.31/6.33*
BAV	24.90	***BAV***	81.12	***BAV***	47.29	***BAV***	-/163.04
QE	1.0098	***QE***	1.0263	***QE***	1.0213	***QE***	-/1.0605
$Ca1O_{12}$				$Ca2O_{12}$			
**Ca1-O12**	2.185(9)	**Ca1-O5**	2.38(3)	**Ca2-O5**	2.20(1)	**Ca2-O8**	2.42(3)
**Ca1-O6**	2.24(1)	**Ca1-O9**	2.40 (2)	**Ca2-O3**	2.21(1)	**Ca2-O2**	2.35(3)
**Ca1-O1**	2.24 (2)	**Ca1-O13**	2.33(3)	**Ca2-O1**	2.29(3)	**Ca2-O14**	2.44(2)
**Ca1-O14**	2.31(1)	**Ca1-O4**	2.43(3)	**Ca2-O9**	2.299(8)	**Ca2-O11**	2.44(2)
**Ca1-O12**	2.37(3)	**Ca1-O16**	2.42(3)	**Ca2-O10**	2.31(3)	**Ca2-O16**	2.52(2)
**Ca1-O8**	2.40(2)	**Ca1-O4**	2.686(8)	**Ca2-O9**	2.34(2)	**Ca2-O10**	2.59(2)
<Ca1-O>	2.37	***Volume***	30.83	***<Ca2-O>***	2.37	***Volume***	31.20
